# Latent Profile Analysis of Resilience, Peer Victimization, and Friendship Quality Configurations Among Chinese Adolescents: Identifying Four Subgroups and Their Self-Injury Prevalence

**DOI:** 10.3390/bs16091538

**Published:** 2026-09-01

**Authors:** Zhuo-Yan Zhang, Rong Xv, Yu-Shuang Chen, Ling-Li Yue, Hai-Shan Huang

**Affiliations:** 1 School of Nursing, Tongji Medical College, Huazhong University of Science and Technology, Wuhan 430030, China; 2 Nursing Department, Tongji Hospital, Tongji Medical College, Huazhong University of Science and Technology, Wuhan 430030, China

**Keywords:** adolescents, self-injury, latent profile analysis, resilience, peer victimization, verbal victimization

## Abstract

This cross-sectional study aimed to identify distinct subgroups of Chinese adolescents based on resilience, peer victimization, and friendship quality, as well as to describe self-injury prevalence across subgroups. Methods: A survey of 33,821 middle school students (mean age 14.3 years; 49.5% male) assessed resilience (RSCA), peer victimization (MPVS), friendship quality (FQQ), depression (PHQ-9), anxiety (GAD-7), and psychological pain (TDPPS). Latent profile analysis (LPA) identified subgroups using 11 indicators; logistic regression examined self-injury associations. Results: Four profiles emerged: High Resource–Low Victimization (85.3%), Moderate Resource–Moderate Victimization (7.6%), Moderate Resource–Polyvictimization (2.1%), and Moderate Resource–Verbal Victimization (5.0%). Self-injury prevalence differed significantly across groups (18.1%, 47.5%, 56.3%, and 44.8%; *p* < 0.001). The Verbal Victimization group had the highest adjusted odds of self-injury (OR = 1.48, 95% CI: 1.31–1.68), followed by Moderate Victimization (OR = 1.38) and Polyvictimization (OR = 1.21). Psychological pain was the strongest correlate (OR = 2.41, 95% CI: 2.30–2.52). Conclusion: Heterogeneous resource–victimization profiles exist among adolescents. Verbal victimization—despite its otherwise moderate resources—was associated with the highest adjusted odds of self-injury, challenging conventional victimization hierarchies. Causal inferences are precluded by the cross-sectional design.

## 1. Introduction

Self-injury, encompassing non-suicidal self-injury (NSSI) and suicide attempts, has emerged as a major public health concern among adolescents globally (Kim et al., 2025; Ma et al., 2025). Beyond causing direct physical harm, self-injurious behavior is one of the strongest prospective correlates of future suicide attempts (Hawton et al., 2012). Self-injury is not a homogeneous phenomenon; substantial individual differences exist in its manifestations, functional motivations, and underlying psychosocial risk factors (Arbuthnott & Lewis, 2015; Calvete et al., 2022). Traditional variable-centered approaches, which examine linear relationships between single or few risk factors and self-injury, struggle to capture the complex heterogeneous patterns formed by the interaction of multiple individual characteristics. In recent years, person-centered approaches such as latent profile analysis (LPA) have offered new perspectives for understanding this heterogeneity, enabling the identification of adolescent subgroups with distinct characteristic combinations based on multidimensional indicators and the examination of differences across these subgroups on outcome variables (Yang et al., 2024).

According to the interpersonal psychological theory of suicide and the ideation-to-action framework, the occurrence of self-injury is theorized to reflect the dynamic interplay between external interpersonal risks and internal personal resources (Janssens et al., 2024; Etherson et al., 2025). These theoretical frameworks typically assume that risk and protective factors are uniformly distributed across individuals. However, adolescents may differ in how these factors combine and interact, suggesting that person-centered approaches like LPA—which identify subgroups with distinct factor configurations—can complement variable-centered tests by revealing heterogeneity in patterns of vulnerability and resources that linear models may obscure. Regarding external risks, school bullying—one of the most prevalent stressors during adolescence—has been consistently associated with self-injurious behavior in multiple studies (Drubina et al., 2023). Beyond these external risks, interpersonal resources—particularly friendship quality—may play a protective role.

Friendship quality—encompassing companionship, intimacy, and emotional support (Parker & Asher, 1993)—is theorized to buffer victimization effects through direct and buffering pathways (Lucas-Molina et al., 2022), consistent with the stress-buffering model (Wang et al., 2021), though most studies have used variable-centered approaches. To our knowledge, no person-centered study has simultaneously integrated friendship quality with resilience and peer victimization to identify how these three domains configure into distinct subgroups. This gap limits understanding of whether friendship quality is universally protective or only benefits specific profiles—a distinction critical for targeted intervention design. Regarding personal resources, resilience—encompassing dimensions such as goal planning, emotional regulation, positive cognition, interpersonal assistance, and family support—represents an internal psychological capital that enables adolescents to cope with adversity and may be descriptively associated with lower likelihood of stress translating into self-injurious behavior (X. Guo et al., 2024). However, previous research has rarely integrated these three categories of factors simultaneously within a unified framework, and there is a particular lack of person-centered investigations revealing heterogeneous subgroups formed by different configurations of vulnerability and resources. The specific research questions guiding this study were: (1) How do resilience, peer victimization, and friendship quality configure into distinct subgroups among Chinese adolescents? (2) What are the sociodemographic and psychological characteristics of each subgroup? (3) How does self-injury prevalence differ across these subgroups in cross-sectional analyses?

To address these research gaps, the present study employs latent profile analysis using 11 specific indicators—the five dimensions of the Resilience Scale for Chinese Adolescents (RSCA), the victimization dimensions of the Multidimensional Peer Victimization Scale (MPVS), and the total score of the Friendship Quality Questionnaire (FQQ)—to descriptively identify distinct subtypes based on “internal resource–external risk–interpersonal support” configurations among Chinese adolescents. After identifying subgroups with distinct psychosocial profiles, we use binary logistic regression (with past-year self-injury as the outcome) to compare self-injury prevalence across these latent profiles. This descriptive approach clarifies which configuration patterns are associated with higher self-injury prevalence, providing an empirical foundation for developing targeted, subgroup-specific prevention strategies. No causal or developmental inferences are drawn from the cross-sectional data.

## 2. Method

### 2.1. Participants and Sample Size

This study employed a multi-center cross-sectional survey design. Using random cluster sampling, we randomly selected two junior high schools and two senior high schools from each of the seven central urban districts of Wuhan (14 junior and 14 senior high schools total, 28 schools overall). All students enrolled in these schools were invited to participate. Of 36,427 eligible students, 33,821 completed valid questionnaires (response rate = 92.8%). Incomplete questionnaires (*n* = 2606) were excluded prior to analysis. Data collection was conducted in December 2025.

Inclusion criteria: ① Adolescents aged 12 to 18 years (inclusive); ② the student understood the study purpose and voluntarily provided consent to participate in the assessment; ③ able to understand Chinese and possessing the basic comprehension skills necessary to complete standardized questionnaires; ④ able to complete the baseline assessment questionnaire within the scheduled school time.

Exclusion criteria: ① Explicit refusal to participate; ② presence of severe intellectual disabilities, cognitive dysfunctions, or significant sensory impairments that would preclude comprehension of the assessment content or effective communication; ③ being in an acute phase of a severe mental illness or acute physical condition at the time of assessment, rendering cooperation impossible; ④ experience of a major traumatic event within one week prior to the assessment, with the assessor judging the individual’s current emotional state as extremely unstable, such that immediate assessment might cause secondary harm or yield inaccurate results; ⑤ recent receipt of professional intervention.

This study was reported in accordance with the STROBE guidelines for cross-sectional studies. The data underlying this study are not publicly available due to ethical restrictions. Access can be requested from the corresponding author with approval from our Ethics Committee.

### 2.2. Ethics

This study was performed in accordance with the guidelines of the Declaration of Helsinki and approved by the Institutional Review Board of Tongji Hospital, Tongji Medical College, Huazhong University of Science and Technology (TJ-IRB202510009). As the study involved adolescents aged 12–18 years, the ethics committee approved a dual-consent process: written informed consent was obtained from a parent or legal guardian, and written informed consent was also obtained from each adolescent participant themselves (including those aged 12–15 and 16–18). These procedures were deemed appropriate given the non-invasive nature of the survey, the adolescents’ capacity to understand the study, and the importance of respecting both parental oversight and adolescent autonomy. The IRB approval date was 29 September 2025. The survey was anonymous. After questionnaire completion, participants were provided with contact information for school counselors and mental health resources.

### 2.3. Measurements and Variable Definition

Demographic Information Questionnaire: This collected basic information including gender, age, and family parenting style.

Questionnaires:

(1) Perceived Stress Scale (CPSS): The CPSS assesses the intensity of an individual’s subjective perception of stress related to life events. It comprises 14 items across two dimensions: tension and loss of control. The scale employs a 5-point Likert scoring system, with higher scores indicating greater perceived stress levels (Cohen et al., 1983; Cortina, 1993). The Cronbach’s α coefficients for the two dimensions were 0.823 and 0.863, respectively, indicating good reliability. For the present study, CPSS scores were transformed to a 2–10 metric for reporting (mean item score × 2) to improve readability alongside other variables. All statistical analyses used the raw total scores (14–70).
(2) Emotional State Assessment: Emotional state was evaluated using two scales: the Patient Health Questionnaire-9 (PHQ-9) and the Generalized Anxiety Disorder-7 (GAD-7). The PHQ-9 screens for depression severity, with the following scoring criteria: 0–4 = no depression; 5–9 = mild depression; 10–14 = moderate depression; 15–19 = moderately severe depression; 20–27 = severe depression (Kroenke et al., 2001). The GAD-7 assesses the frequency of anxiety symptoms, with scoring: 0–4 = normal; 5–9 = mild anxiety; 10–14 = moderate anxiety; 15–21 = severe anxiety (Spitzer et al., 2006). The Cronbach’s α coefficients were 0.890 for the PHQ-9 and 0.918 for the GAD-7, indicating good reliability.
(3) Resilience Scale for Chinese Adolescents (RSCA): This scale comprises five dimensions: goal planning, emotional regulation, positive cognition, interpersonal assistance, and family support. It uses a 5-point Likert scoring system, with higher scores indicating greater psychological resilience (Hu & Gan, 2008). The Cronbach’s α coefficient for this scale was 0.646. Although this reliability value is slightly below the conventionally recommended threshold of 0.70, it remains acceptable in the context of latent profile analysis and large sample sizes. The model fit indices and entropy values obtained in subsequent analyses indicated that the latent profile structure identified using this measurement tool demonstrated good classification clarity and statistical reliability (Tein et al., 2013). Subscale-level reliability coefficients were not available for the present sample; this is acknowledged as a limitation.
(4) Three-Dimensional Psychological Pain Scale (TDPPS): This scale consists of 17 items across three dimensions: pain experience, pain arousal, and pain avoidance (Li et al., 2014). It employs a 5-point Likert scoring system, with higher total scores indicating greater levels of psychological pain. The Cronbach’s α coefficient for this scale was 0.952, indicating excellent reliability.
(5) Friendship Quality Questionnaire (FQQ): This questionnaire comprises 18 items rated on a 5-point Likert scale (1 = “completely untrue,” 5 = “completely true”), with higher scores reflecting better friendship quality (Parker & Asher, 1993). The Cronbach’s α coefficient for this questionnaire was 0.905, indicating good reliability.
(6) Multidimensional Peer Victimization Scale (MPVS): This scale consists of 25 items across five dimensions—physical victimization, social manipulation, verbal victimization, attacks on property, and cyber victimization—reflecting the extent of school bullying (Mynard & Joseph, 2000; Betts et al., 2015). Each item uses an 8-point Likert scale to measure the degree of bullying currently experienced. The Cronbach’s α coefficient for this scale was 0.790, indicating acceptable reliability. This study used a validated Chinese adaptation of the MPVS with an 8-point frequency response format (Chen et al., 2009; W. X. Zhang et al., 2009), which has been shown to have acceptable psychometric properties in Chinese adolescent samples.
(7) Self-Injury Outcome. Self-injury was assessed using a single item adapted from the Chinese version of the Adolescent Self-Injury Questionnaire: “In the past 12 months, have you deliberately hurt yourself (e.g., cutting, burning, hitting, scratching) without a fatal intent?” Response options were “No” (coded 0) and “Yes” (coded 1). The item did not differentiate between non-suicidal self-injury and suicide attempts, as the study was designed for community screening rather than clinical diagnosis. Participants who responded “Yes” were coded as having engaged in self-injury in the past year. This binary measure precludes analysis of frequency, method, or severity, which is acknowledged as a limitation.

### 2.4. Statistical Analysis

Data were double-entered using EpiData 3.1 and checked for consistency. In cases of data inconsistency, original questionnaires were consulted promptly for verification. Statistical analyses were performed using SPSS 25.0 and Mplus 8.3. Missing values (<5% for all variables) were handled using multiple imputation with 20 imputed datasets for the logistic regression analysis. For the latent profile analysis, full-information maximum likelihood (FIML) was employed. Sensitivity analyses using complete-case analysis yielded substantively identical results. Reverse-scored items were transformed prior to analysis. All 11 indicators were standardized to z-scores prior to analysis because they were measured on different scales. Latent profile analysis (LPA) was conducted using the five dimensions of the Resilience Scale for Chinese Adolescents (RSCA), the five subscales of the Multidimensional Peer Victimization Scale (MPVS), and the total score of the Friendship Quality Questionnaire (FQQ) as indicator variables. Model estimation employed maximum likelihood estimation with robust standard errors (MLR). To ensure the global maximum was reached, models were estimated using 500 random starts and 200 final-stage optimizations (Mplus STARTS = 500,200). The selected four-class solution was consistently replicated across multiple random starts. The optimal number of latent classes was determined by comprehensively comparing multiple fit indices, including the Akaike Information Criterion (AIC), Bayesian Information Criterion (BIC), sample size-adjusted BIC (aBIC), Lo–Mendell–Rubin likelihood ratio test (LMR-LRT), bootstrap likelihood ratio test (BLRT), and entropy. Individuals were assigned to their most likely class based on posterior probabilities. Given that most continuous variables exhibited skewed distributions, group comparisons were conducted using Kruskal–Wallis H tests and Mann–Whitney U tests, while categorical variables were analyzed using chi-square tests. Subsequently, binary logistic regression analysis was performed to examine the associations between latent profile membership and self-injurious behavior, calculating odds ratios (ORs) and their 95% confidence intervals (CIs). The analyses controlled for potential confounders including age, gender, parental marital relationship, parenting style, subjective family economic status, and academic ranking. The goal of the LPA was purely descriptive: to identify subgroups of adolescents who share similar configurations of resilience, victimization, and friendship quality, without claiming any developmental or causal process.

## 3. Result

### 3.1. Latent Profile Analysis Results

Latent profile analysis was conducted specifying one- to seven-class solutions. Fit indices for all models are presented in Table 1. To determine the optimal number of classes, we considered statistical fit, parsimony, theoretical interpretability, and class stability. Although information criteria (AIC, BIC, aBIC) continued to decrease beyond four classes, the decrements were progressively smaller, and the five-class solution produced an additional class that essentially split the largest profile (85.3%) into two subgroups differing only marginally in indicator scores, without yielding conceptually distinct clinical profiles. The four-class solution achieved the best balance: entropy was excellent (0.985); all classes were substantively meaningful; and the smallest class (2.08%, *n* = 704) remained sufficiently large for stable estimation given the total sample size. Average posterior probabilities for the four-class solution ranged from 0.958 to 0.998 across classes (Class 1: 0.994, Class 2: 0.958, Class 3: 0.998, Class 4: 0.984), indicating excellent classification certainty. In contrast, the five-class solution, despite slightly lower BIC, produced profiles that were less interpretable and offered no additional theoretical insight. We acknowledge that the BLRT and VLMR tests for the 1- to 2-class comparison were non-significant (*p* = 0.3328 and *p* = 0.3315). This indicates that a simple binary split does not adequately capture the heterogeneity in the data. The four-class solution was retained because it revealed qualitatively distinct and theoretically coherent profiles beyond a low-risk/high-risk dichotomy, and the solution was replicated across multiple random starts (see Methods). We therefore retained the four-class model as the most parsimonious and clinically meaningful solution.

### 3.2. Profile Characteristics

**Figure 1:**
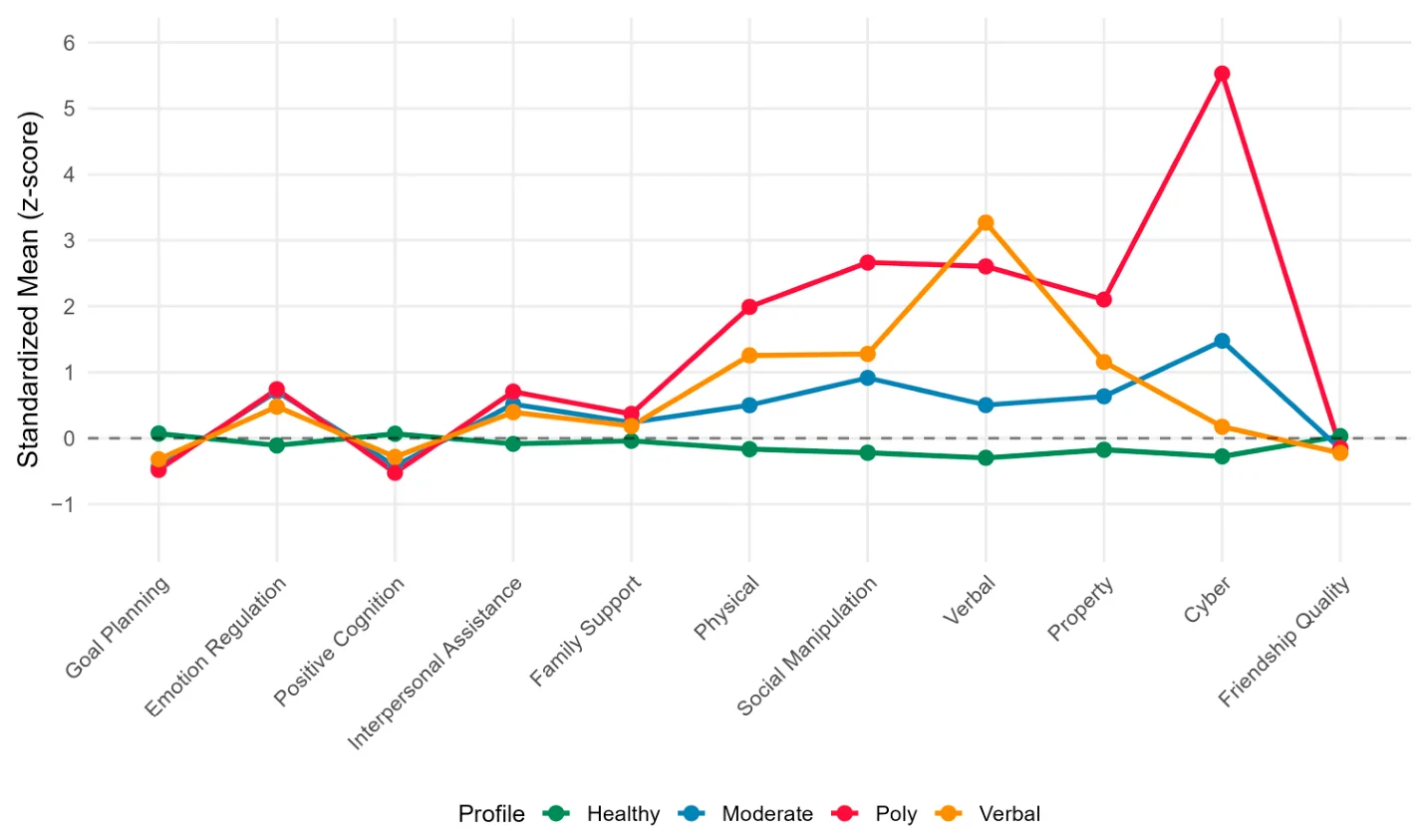
Characteristic Profiles of the Four Latent Classes. The y-axis represents standardized mean scores (z-scores) for each indicator variable. Standardization allows direct visual comparison across indicators measured on different scales (5-point for RSCA and FQQ, 8-point for MPVS). Higher scores indicate higher levels of the respective construct relative to the sample mean. RSCA dimensions: Goal Planning, Emotional Regulation, Positive Cognition, Interpersonal Assistance, Family Support; MPVS dimensions: Physical Victimization, Social Manipulation, Verbal Victimization, Attacks on Property, Cyber Victimization; FQQ: Friendship Quality total score. Profiles: Healthy (*n* = 28,860), Moderate (*n* = 2583), Poly (*n* = 704), Verbal (*n* = 1674). The dotted horizontal line at y = 0 represents the sample mean reference line.

The characteristic distribution of the four latent classes is shown in Figure 1. Profile 1 was named the “High Resource–Low Victimization Group”, comprising 85.33% of the sample. This group had a sum of mean scores across the five resilience dimensions of 15.484 (dimension mean range: 2.756–3.624), a mean friendship quality score of 3.433, and a sum of mean scores across the five peer victimization dimensions of 6.216 (dimension mean range: 1.066–1.343). Although its total resilience score was slightly lower than that of other groups, this profile demonstrated the highest friendship quality and the lowest levels of victimization, hence the designation “High Resource.” The label “High Resource” reflects this group’s relatively high friendship quality and resilience on standardized indicators, rather than absolute resilience scores.

Profile 2 was named the “Moderate Resource–Moderate Victimization Group”, comprising 7.64% of the sample. This group had a sum of mean resilience dimension scores of 15.682 (dimension mean range: 2.915–3.386), a mean friendship quality score of 3.293, and a sum of mean victimization dimension scores of 13.552 (dimension mean range: 1.881–2.885). This profile exhibited moderate levels of both protective factors and victimization exposure.

Profile 3 was named the “Moderate Resource–Polyvictimization Group”, comprising 2.08% of the sample. This group had a sum of mean resilience dimension scores of 15.752 (dimension mean range: 2.875–3.419), a mean friendship quality score of 3.299, and a substantially higher sum of mean victimization dimension scores of 26.234 (dimension mean range: 3.522–7.156). Notably, this profile showed extremely high scores for verbal victimization (6.126) and cyber victimization (7.156), reflecting a pattern of co-occurring multidimensional victimization.

Profile 4 was named the “Moderate Resource–Verbal Victimization Group”, comprising 4.95% of the sample. This group had a sum of mean resilience dimension scores of 15.645 (dimension mean range: 2.966–3.295), a mean friendship quality score of 3.243, and a sum of mean victimization dimension scores of 17.865 (dimension mean range: 1.539–7.232). Critically, this profile exhibited the highest verbal victimization score across all four groups (7.232) while showing the lowest cyber victimization score (1.539), indicating a distinct pattern dominated specifically by verbal victimization.

### 3.3. Demographic and Psychological Characteristics Across Latent Profiles

Significant differences were observed across the four groups in family environment, mental health, and victimization experiences, as shown in Table 2. The “High Resource–Low Victimization Group” demonstrated the most optimal family functioning, the lowest levels of depression and anxiety, and a self-injury prevalence of only 18.1%, reflecting a descriptive pattern consistent with protection. In contrast, the “Moderate Resource–Polyvictimization Group” exhibited the most disadvantaged profile, with the highest proportions of parental discord and neglectful parenting, median depression (20.0) and anxiety (17.0) scores reaching at least moderate levels, and self-injury prevalence escalating to 56.3%. Gender distribution showed specific patterns: the Verbal Victimization Group had the highest proportion of males (65.1%), whereas the Moderate Victimization Group was predominantly female (61.2%). As victimization types diversified and protective factors weakened, adolescents’ psychological distress (PHQ-9 and GAD-7 scores) increased in a stepwise manner, while psychological pain scores (TDPPS) concurrently increased, as expected given the greater adversity in these profiles. This pattern indicates that the cumulative harm of multiple victimization experiences on adolescent development substantially exceeds that of single victimization forms, and the descriptive association between family environment and self-injury prevalence was less pronounced among adolescents with frequent victimization in this sample.

### 3.4. Logistic Regression Analysis of Factors Associated with Self-Injury Across Latent Profiles

To examine the cross-sectional association between latent profile membership and self-injurious behavior in adolescents, binary logistic regression analysis was conducted with the healthiest group (Group 1: High Resource–Low Victimization) as the reference, while controlling for demographic variables, family factors, and psychological states. The Omnibus test indicated that the model was statistically significant (χ^2^ = 9404.22, df = 17, *p* < 0.001), and the Nagelkerke R^2^ value was 0.37, indicating an acceptable model fit for the logistic regression (pseudo-R^2^ measures in logistic regression should not be interpreted as proportion of explained variance). Classification accuracy improved from 77.5% in the null model to 82.5% in the full model, with a sensitivity (accuracy for classifying “presence” of self-injury) of 41.6% and a specificity (accuracy for classifying “absence”) of 94.3%.

As shown in Table 3, compared to the reference Group 1, Group 2 (Moderate Resource–Moderate Victimization) had 1.376 times higher odds of self-injury (OR = 1.376, *p* < 0.001), Group 3 (Moderate Resource–Polyvictimization) had 1.214 times higher odds (OR = 1.214, *p* = 0.045), and Group 4 (Moderate Resource–Verbal Victimization) had 1.483 times higher odds (OR = 1.483, *p* < 0.001). These cross-sectional associations indicate that, relative to the High Resourc–-Low Victimization group, all three other profiles showed significantly elevated odds of self-injury, with Group 4 demonstrating the highest odds. These findings should not be interpreted as evidence of causal risk processes. Importantly, given the large sample size, we emphasize effect sizes (odds ratios and their 95% confidence intervals) over *p*-values. For example, the verbal victimization group had 1.48 times higher odds of self-injury compared to the healthy group, with a narrow confidence interval (1.31–1.68), indicating a practically meaningful association.

To examine whether psychological states (depression, anxiety, and psychological pain) functioned as mediators rather than confounders, we re-ran the logistic regression model excluding PHQ-9, GAD-7, and TDPPS. In this unadjusted model, the ORs for the three victimization profiles were substantially larger: Moderate Victimization OR = 3.30 (95% CI: 3.00–3.63), Polyvictimization OR = 3.41 (95% CI: 2.85–4.09), and Verbal Victimization OR = 2.72 (95% CI: 2.43–3.04). After including psychological states as covariates, these ORs were attenuated to 1.29 (95% CI: 1.16–1.45), 0.78 (95% CI: 0.63–0.97), and 1.18 (95% CI: 1.04–1.35), respectively. This marked attenuation—particularly for the Polyvictimization group (from 3.41 to 0.78)—indicates that psychological pain and affective symptoms largely mediate the association between polyvictimization and self-injury, rather than acting as independent confounders. These results are presented in Supplementary Table S1. Given the cross-sectional design, these patterns should be interpreted as descriptive and hypothesis-generating.

Psychological pain showed the strongest cross-sectional association with self-injury (OR = 2.405, *p* < 0.001), followed by depression severity (OR = 1.100, *p* < 0.001). However, because these variables are measured on different scales (TDPPS as a mean item score on a 0–5 scale, PHQ-9 as a 0–27 total score, and GAD-7 as a 0–21 total score), direct comparison of their unstandardized ORs is not appropriate for assessing relative strength. After standardizing all continuous predictors to permit relative comparison (per one standard deviation increase), psychological pain showed the strongest association with self-injury (standardized OR = 2.82, 95% CI: 2.69–2.96), followed by depression (standardized OR = 1.74, 95% CI: 1.63–1.86) and anxiety (standardized OR = 1.07, 95% CI: 0.99–1.16). This suggests that subjectively experienced psychological pain and depressive symptoms are cross-sectionally associated with self-injury (Tang et al., 2025; J. Guo et al., 2025). Male gender (OR = 1.265), poorer parental marital relationship (OR = 1.080), maladaptive parenting style (OR = 1.077), lower subjective family economic status (OR = 1.056), and lower academic ranking (OR = 1.045) were all significantly associated with higher odds of self-injury in this cross-sectional analysis. Each one-year increase in age was associated with lower odds of self-injury (OR = 0.858, *p* < 0.001). The effects of only-child status, residence, parental education levels, and perceived stress were not statistically significant (*p* > 0.05).

## 4. Discussion

### 4.1. Summary of Main Findings

Using latent profile analysis based on 11 indicators across three core dimensions—psychological resilience, peer victimization, and friendship quality—this cross-sectional study identified four distinct subgroups among 33,821 Chinese adolescents: the “High Resource–Low Victimization Group” (85.33%), the “Moderate Resource–Moderate Victimization Group” (7.64%), the “Moderate Resource–Polyvictimization Group” (2.08%), and the “Moderate Resource–Verbal Victimization Group” (4.95%). Significant differences in the prevalence of self-injury were observed across the profiles, and profile membership remained significantly associated with self-injury prevalence after controlling for covariates. These findings are descriptive; they do not imply causal or developmental processes.

### 4.2. Characteristics and Theoretical Implications of Four Latent Profiles

High Resource–Low Victimization Group. As the predominant subgroup, this profile was characterized by relatively high levels of psychological resilience, the highest friendship quality, and the lowest levels of peer victimization. The self-injury prevalence in this group was only 18.1%, accompanied by the lowest levels of depression and anxiety. These findings align with the stress-buffering model, which posits that ample internal psychological resources and external interpersonal support can effectively buffer the negative impact of stress on mental health (Lucas-Molina et al., 2022; Leng et al., 2025). The existence of this large, relatively healthy subgroup suggests that most adolescents function within a supportive psychosocial environment, providing a realistic foundation for promotion-oriented school mental health education (McEvoy et al., 2025). Recent studies have also confirmed that high psychological resilience significantly reduces the risk of self-injury among adolescents (Haham et al., 2026; D. Zhang et al., 2025).

Moderate Resource–Moderate Victimization Group. This group exhibited psychological resilience levels similar to the high-resource group but experienced moderate levels of victimization. Their self-injury prevalence rose to 47.5%, with median depression (18.0) and anxiety (14.0) scores markedly elevated. This pattern indicates that when psychological resources are comparable, differences in victimization experiences were descriptively associated with mental health outcomes in this sample, consistent with the cumulative risk hypothesis: once victimization reaches a certain intensity, its association with adverse mental health outcomes may outweigh the potential benefits of protective factors (Calear et al., 2025).

Moderate Resource–Polyvictimization Group. Although psychological resilience levels in this group were close to those of the previous groups, they encountered extremely high frequencies of multidimensional victimization, particularly verbal and cyber victimization. Their self-injury prevalence reached 56.3%, and they had the highest psychological pain scores, again supporting the cumulative risk hypothesis. This group also had the highest proportions of neglectful parenting (12.2%) and poor parental marital relationships (23.1%), suggesting that family system dysfunction may be an important backdrop for polyvictimization (McEvoy et al., 2025).

Moderate Resource–Verbal Victimization Group. This profile displayed a unique victimization pattern: the highest verbal victimization score among all four groups combined with the lowest cyber victimization score. The self-injury prevalence was 44.8%, and males constituted 65.1% of this group. The existence of this profile challenges the traditional hierarchical view that “physical victimization > verbal victimization” and highlights the independent harm of verbal victimization. The everyday, persistent nature of verbal aggression and its direct attack on self-esteem may more readily trigger negative cognitions associated with self-injury (Calear et al., 2025). The high proportion of males may relate to gender socialization; boys are often expected to be “tough” and may suppress emotional distress, which can instead be expressed through externalizing behaviors such as self-injury (Álvarez-Voces et al., 2025).

### 4.3. Verbal Victimization as a Distinct Profile with Elevated Adjusted Odds

The finding that the “Verbal Victimization Group” showed the highest adjusted odds of self-injury (OR = 1.483) has important implications. Verbal victimization, though pervasive, is often overlooked by educators and parents because it leaves no visible scars. However, this study demonstrates that when verbal victimization becomes the dominant form of peer abuse, its adjusted association with self-injury was stronger than that of polyvictimization in this sample. This may be attributable to two characteristics of verbal victimization: first, its chronic and pervasive nature makes it difficult to avoid through physical separation, unlike physical bullying; second, it directly attacks self-concept and self-esteem, potentially more effectively activating the negative cognitive schemas linked to self-injury (Yun et al., 2019; Fang et al., 2025). The predominance of males in this profile also underscores the need to attend to male adolescents’ vulnerability to verbal victimization. Due to societal expectations, males’ distress is often suppressed or overlooked, and they may be more inclined to use avoidance and suppression strategies when facing verbal bullying, which in turn is associated with higher odds of self-injury (Moloney et al., 2024).

### 4.4. Profile-Specific Associations and Overadjustment

With the ‘High Resource–Low Victimization Group’ as a reference, the verbal victimization group had the highest adjusted odds (OR = 1.483), followed by the moderate victimization group (OR = 1.376)—yet the polyvictimization group, despite having the highest crude prevalence (56.3%), showed the smallest adjusted OR (OR = 1.214). This pattern appears to reflect statistical overadjustment rather than a genuine protective effect. When psychological states (PHQ-9, GAD-7, TDPPS) were removed from the model, the OR for polyvictimization increased from 1.21 to 3.41 (95% CI: 2.85–4.09), while the ORs for the moderate and verbal victimization groups increased to 3.30 and 2.72, respectively. The disproportionate attenuation for the polyvictimization group (the group with the highest TDPPS scores, median = 3.6) suggests that their elevated psychological pain accounts for a substantial portion of their cross-sectional self-injury odds, consistent with the ideation-to-action framework in which external adversities influence self-injury through internal psychological states (Ribeiro et al., 2016). This pattern is descriptively consistent with the theoretical role of psychological pain and affective symptoms as potential mediators of the victimization–self-injury relationship. It should be noted that the confidence intervals for the adjusted ORs of the Verbal (1.31–1.68) and Polyvictimization (1.01–1.47) groups overlapped substantially; no formal statistical comparison between these ORs was conducted. Thus, the relative ordering should be interpreted cautiously and treated as descriptive rather than inferential. Notably, the psychological resilience score of the polyvictimization group (15.75) did not differ significantly from that of the high-resource group (15.48), suggesting that although these adolescents faced multiple adversities, their psychological resilience had not completely collapsed, potentially attenuating the victimization–self-injury association. This observation aligns with the cognitive vulnerability-stress model: when an individual’s psychological resources match the intensity of stressors, a pattern that may be descriptively consistent with residual protective influences (Wei et al., 2022).

Psychological pain showed the strongest cross-sectional association with self-injury (OR = 2.405), followed by depression severity (OR = 1.100). This indicates that, regardless of profile membership, subjectively experienced psychological pain and depressive symptoms are proximal correlates associated with self-injurious behavior in this cross-sectional sample (Tang et al., 2025; J. Guo et al., 2025). This finding supports the ideation-to-action framework, which posits that external adversities are associated with self-injury through internal psychological states such as pain and depression (Ribeiro et al., 2016).

Demographic factors also showed significant cross-sectional associations with self-injury prevalence. Males had higher odds of self-injury than females (OR = 1.265), a finding that diverges from some clinical studies but aligns with large-scale epidemiological surveys (Ren et al., 2025). This discrepancy may be due to our community-based sample of general middle school students and the use of a binary measure (“any self-injury in the past year”) rather than focusing on repetitive self-injury. Additionally, recent cross-sectional surveys have noted rising rates of self-reported self-injury among male adolescents in Asian cultural contexts, possibly related to shifting gender role expectations (Moloney et al., 2024; Yin et al., 2025). However, these interpretations remain speculative without longitudinal data.

Age showed a significant negative association with self-injury: each one-year increase in age was associated with lower odds of self-injury (OR = 0.858). This age-related decline may reflect improvements in cognitive maturity, emotion regulation capabilities, and the stabilization of social support networks. Family factors showed consistent cross-sectional associations with self-injury odds, underscoring the potential role of the family system in adolescent mental health. In particular, the associations of neglectful parenting (OR = 1.077) and poor parental marital relationships (OR = 1.080) resonate with the core tenet of interpersonal theory: that adverse interpersonal environments—including those within the family—are important distal antecedents of self-injurious behavior (Wang et al., 2025). A 2025 review concluded that improving parent–child communication and family functioning may be associated with lower self-injury incidence (Ojala et al., 2026).

### 4.5. Critical Evaluation of Theoretical Frameworks

Our descriptive findings are broadly consistent with the ideation-to-action framework in that external adversities were cross-sectionally associated with self-injury (Ribeiro et al., 2016), and this association was substantially attenuated when internal psychological states were considered. This pattern supports the framework’s premise that external adversities are cross-sectionally associated with self-injury through internal mechanisms. However, the cross-sectional design prevents testing the temporal ordering implied by the model. More importantly, our identification of a distinct “verbal victimization” profile—which showed a stronger adjusted association with self-injury than polyvictimization—extends the framework by suggesting that the type of victimization may matter more than the number of victimization types, a distinction not explicitly captured by current cumulative risk models (Calear et al., 2025). This finding invites theoretical refinement: future models should account for the specific content of interpersonal adversity, not merely its cumulative burden.

Our descriptive findings may also be situated within the broader developmental literature on childhood adversity and early maladaptive schemas. Childhood maltreatment and peer victimization have been shown to contribute to the development of dysfunctional schemas—such as mistrust, defectiveness, and emotional deprivation—which in turn are associated with emotional dysregulation, self-injurious behaviors, and, in some adolescents, aggressive or violent behaviors (Rada et al., 2025; May et al., 2022). Early maladaptive schemas have been directly linked to self-injury thoughts and behaviors in adolescent samples (Saarijärvi et al., 2023). The verbal victimization profile identified in our sample may represent a group in which such schema-based vulnerabilities are particularly salient, as verbal aggression directly attacks self-worth and social acceptance. Protective factors—including family support, positive peer relationships, and emotion regulation skills—have been consistently associated with lower risk of both self-harm and externalizing problems (Ciurbea et al., 2025). Integrating these developmental and risk-protection frameworks suggests that the identified profiles may not only inform self-injury prevention but also point to broader intervention targets for emotional and behavioral difficulties in adolescence.

## 5. Limitation

Several limitations should be acknowledged. First, the cross-sectional design precludes causal or developmental inferences; all findings are descriptive and hypothesis-generating. This is the central limitation, and the primary contribution is generating testable hypotheses for future longitudinal research. Second, all variables relied on self-report, which may introduce common method bias; multi-informant approaches are needed. Third, the RSCA showed modest reliability (α = 0.646), which is acceptable for LPA with large samples but may affect profile precision. Fourth, the urban Wuhan sample limits generalizability to rural areas or other provinces. Fifth, self-injury was measured dichotomously (presence/absence in the past year), preventing analysis of frequency or function. Sixth, the small polyvictimization class (2.1%) and the overall profile solution were not formally validated using split-sample replication or bootstrap procedures. Although the four-class solution was replicated across multiple random starts and demonstrates good entropy, the stability and generalizability of these profiles should be confirmed in independent samples. Readers are therefore cautioned against overinterpreting the specific ordering of the profiles. Seventh, classification uncertainty and school-level nesting were not modeled; future studies should consider BCH correction and cluster-robust standard errors.

## 6. Conclusions

This cross-sectional study identified four distinct adolescent subgroups based on resilience, peer victimization, and friendship quality. The verbal victimization profile showed the highest adjusted odds of self-injury, whereas the polyvictimization group had the highest unadjusted prevalence (56.3%). These descriptive profiles are consistent with stress-buffering and cumulative risk frameworks, though causal inferences are precluded by the cross-sectional design. Psychological pain showed the strongest cross-sectional association with self-injury, and male adolescents in the verbal victimization profile exhibited elevated vulnerability. The findings support a descriptive profiling approach for targeted, tiered prevention, but longitudinal research with multi-informant methods is needed to test predictive validity and developmental pathways.

### Relevance to Clinical Practice

Clinicians may benefit from assessing verbal victimization, as it showed the strongest adjusted association with self-injury in this cross-sectional study, despite being often overlooked, particularly in males. Psychological pain was the strongest cross-sectional correlate, suggesting that distress-focused interventions such as emotion regulation training may be beneficial. Family factors—neglectful parenting and parental discord—may warrant family involvement, though this requires further testing. Early adolescence may represent a developmentally sensitive period for stratified prevention: universal resilience-building for most, targeted victimization-focused interventions for subgroups with elevated victimization exposure, and family-involved treatment for those with the most complex needs, but these recommendations should be evaluated in prospective intervention studies.

## Figures and Tables

**Table 1:** Fit Indices for Latent Profile Analysis Models.

Model	LL	AIC	BIC	aBIC	Entropy	*p*		Class Probabilities (%)
						BLRT	VLMR	
**1**	**−500,363**	**1,000,770.334**	**1,000,955.768**	**1,000,886**				1
2	−467,752	935,571.195	935,857.775	935,749.7	0.989	0.3328	0.3315	7.98/92.02
3	−454,714	909,520.594	909,908.32	909,762.1	0.977	<0.001	<0.001	2.22/9.63/88.15
**4**	**−** **441** **,** **922**	**883** **,** **960.261**	**884** **,** **449.134**	**884** **,** **264.8**	**0.985**	**<0.001**	**<0.001**	**2.08/4.95/7.64/85.33**
5	−432,118	864,375.758	864,965.776	864,743.3	0.874	<0.001	<0.001	2.06/4.85/6.67/39.41/47.01
6	−422,032	844,227.956	844,919.121	844,658.5	0.896	<0.001	<0.001	2.06/2.26/4.84/6.65/36.48/47.71
7	−414,557	829,301.251	830,093.562	829,794.8	0.904	<0.001	<0.001	1.67/2.05/2.23/3.85/6.49/36.24/47.45

LL = log-likelihood; AIC = Akaike Information Criterion; BIC = Bayesian Information Criterion; aBIC = sample size-adjusted BIC; BLRT = bootstrap likelihood ratio test; VLMR = Vuong-Lo-Mendell-Rubin likelihood ratio test. Entropy values range from 0 to 1, with values > 0.80 indicating good classification accuracy. Significant BLRT and VLMR (*p* < 0.05) suggest that the k-class model fits significantly better than the k-1 class model. The selected four-class model is indicated in bold.

**Table 2:** Comparison of Demographic and Psychological Characteristics Across Latent Profiles.

Variable	Category	High Resource–Low Victimization (*n* = 28,860)	Moderate Resource–Moderate Victimization (*n* = 2583)	Moderate Resource–Polyvictimization (*n* = 704)	Moderate Resource–Verbal Victimization (*n* = 1674)	Test Statistic	df	*p*
Gender	Male	15,184 (52.6)	1001 (38.8)	374 (53.1)	1089 (65.1)	χ^2^ = 300.217	3	<0.001
	Female	13,676 (47.4)	1582 (61.2)	330 (46.9)	585 (34.9)			
Age (years)	12	3212 (11.1)	256 (9.9)	85 (12.1)	259 (15.5)	χ^2^ = 164.655	18	<0.001
	13	6771 (23.5)	617 (23.9)	193 (27.4)	470 (28.1)			
	14	6772 (23.5)	651 (25.2)	183 (26.0)	431 (25.7)			
	15	4743 (16.4)	449 (17.4)	109 (15.5)	278 (16.6)			
	16	3418 (11.8)	314 (12.2)	71 (10.1)	104 (6.2)			
	17	2788 (9.7)	219 (8.5)	41 (5.8)	103 (6.2)			
	18	1156 (4.0)	77 (3.0)	22 (3.1)	29 (1.7)			
Only Child	Yes	16,628 (57.6)	1380 (53.4)	371 (52.7)	949 (56.7)	χ^2^ = 23.025	3	<0.001
	No	12,232 (42.4)	1203 (46.6)	333 (47.3)	725 (43.3)			
Residence	Urban center	27,628 (95.7)	2451 (94.9)	656 (93.2)	1571 (93.8)	χ^2^ = 38.378	6	<0.001
	Urban–rural fringe	1158 (4.0)	123 (4.8)	40 (5.7)	96 (5.7)			
	Rural	74 (0.3)	9 (0.3)	8 (1.1)	7 (0.4)			
Father’s Education	Primary or below	664 (2.3)	80 (3.1)	22 (3.1)	51 (3.0)	χ^2^ = 90.313	15	<0.001
	Middle school	4602 (15.9)	502 (19.4)	106 (15.1)	327 (19.5)			
	High school/Technical	7054 (24.4)	698 (27.0)	167 (23.7)	400 (23.9)			
	Associate degree	4227 (14.6)	387 (15.0)	96 (13.6)	247 (14.8)			
	Bachelor’s degree	9534 (33.0)	731 (28.3)	225 (32.0)	502 (30.0)			
	Master’s or above	2779 (9.6)	185 (7.2)	88 (12.5)	147 (8.8)			
Mother’s Education	Primary or below	1070 (3.7)	108 (4.2)	28 (4.0)	78 (4.7)	χ^2^ = 96.122	15	<0.001
	Middle school	4663 (16.2)	516 (20.0)	110 (15.6)	322 (19.2)			
	High school/Technical	6819 (23.6)	687 (26.6)	188 (26.7)	372 (22.2)			
	Associate degree	4476 (15.5)	388 (15.0)	98 (13.9)	271 (16.2)			
	Bachelor’s degree	9599 (33.3)	728 (28.2)	200 (28.4)	513 (30.6)			
	Master’s or above	2233 (7.7)	156 (6.0)	80 (11.4)	118 (7.1)			
Parental Marital Relationship	Very good	13,326 (46.2)	672 (26.0)	230 (32.7)	521 (31.1)	χ^2^ = 958.031	12	<0.001
	Good	8548 (29.6)	810 (31.4)	158 (22.4)	514 (30.7)			
	Fair	4514 (15.6)	603 (23.3)	153 (21.7)	339 (20.3)			
	Poor	1350 (4.7)	249 (9.6)	70 (9.9)	145 (8.7)			
	Very poor	1122 (3.9)	249 (9.6)	93 (13.2)	155 (9.3)			
Parenting Style	Democratic	23,650 (82.0)	1553 (60.1)	375 (53.3)	1036 (61.9)	χ^2^ = 1310.782	9	<0.001
	Authoritarian	3676 (12.7)	696 (26.9)	213 (30.3)	446 (26.6)			
	Permissive	566 (2.0)	92 (3.6)	30 (4.3)	71 (4.2)			
	Neglectful	968 (3.4)	242 (9.4)	86 (12.2)	121 (7.2)			
Subjective Family Economic Status	Very poor	298 (1.0)	70 (2.7)	41 (5.8)	46 (2.7)	χ^2^ = 512.066	12	<0.001
	Poor	1030 (3.6)	193 (7.5)	64 (9.1)	146 (8.7)			
	Average	12,118 (42.0)	1210 (46.8)	291 (41.3)	747 (44.6)			
	Good	11,804 (40.9)	894 (34.6)	218 (31.0)	586 (35.0)			
	Very good	3610 (12.5)	216 (8.4)	90 (12.8)	149 (8.9)			
Academic Ranking	Top 10%	6134 (21.3)	448 (17.3)	133 (18.9)	295 (17.6)	χ^2^ = 163.227	12	<0.001
	10–30%	8142 (28.2)	674 (26.1)	180 (25.6)	423 (25.3)			
	30–50%	6845 (23.7)	606 (23.5)	126 (17.9)	370 (22.1)			
	50–70%	5074 (17.6)	532 (20.6)	155 (22.0)	342 (20.4)			
	70–100%	2665 (9.2)	323 (12.5)	110 (15.6)	244 (14.6)			
Self-Injury	No	23,625 (81.9)	1355 (52.5)	308 (43.8)	924 (55.2)	χ^2^ = 2181.152	3	<0.001
	Yes	5235 (18.1)	1228 (47.5)	396 (56.3)	750 (44.8)			
PHQ-9	M (P25, P75)	13.0 (10.0, 16.0)	18.0 (14.0, 22.0)	20.0 (16.0, 26.0)	17.0 (13.0, 22.0)	H = 3105.644	3	<0.001
GAD-7	M (P25, P75)	10.0 (7.0, 13.0)	14.0 (11.6, 18.0)	17.0 (13.0, 23.0)	14.0 (10.0, 19.0)	H = 3034.560	3	<0.001
CPSS	M (P25, P75)	6.0 (5.4, 6.3)	6.0 (5.6, 6.4)	6.0 (5.6, 6.6)	6.0 (5.6, 6.4)	H = 64.309	3	<0.001
TDPPS	M (P25, P75)	1.9 (1.2, 2.7)	3.1 (2.5, 3.6)	3.6 (2.8, 4.2)	2.9 (2.2, 3.6)	H = 3945.548	3	<0.001

Data are presented as n (%) for categorical variables and median (25th percentile, 75th percentile) for continuous variables. CPSS scores are presented as mean item score × 2, yielding a range of 2–10. The original scale comprises 14 items rated on a 5-point Likert scale (raw total score range: 14–70); higher scores indicate greater perceived stress. Effect sizes: Cramer’s V for categorical variables ranged from 0.022 to 0.113; η^2^ for continuous variables ranged from 0.003 to 0.137, indicating small-to-moderate effects. The uniformly significant *p*-values (*p* < 0.001) are largely attributable to the exceptionally large sample size (N = 33,821).

**Table 3:** Binary Logistic Regression Analysis of Self-Injury.

Variable	B	SE	Wald χ2	df	*p*	Exp(B)	95% CI for Exp(B)	
							Lower	Upper
Gender (male = 1)	0.235	0.032	54.804	1	<0.001	1.265	1.188	1.346
Age	−0.154	0.01	229.927	1	<0.001	0.858	0.841	0.875
Only child (yes = 1)	−0.023	0.032	0.496	1	0.481	0.978	0.918	1.041
Residence (urban = 1)	0.07	0.068	1.048	1	0.306	1.072	0.938	1.225
Father’s education level	0.023	0.017	1.928	1	0.165	1.024	0.99	1.058
Mother’s education level	0.027	0.017	2.582	1	0.108	1.027	0.994	1.062
Parental marital relationship	0.077	0.015	27.554	1	<0.001	1.08	1.049	1.111
Parenting style	0.074	0.021	12.989	1	<0.001	1.077	1.034	1.122
Subjective family economic status	0.055	0.022	6.475	1	0.011	1.056	1.013	1.102
Academic ranking	0.044	0.013	12.248	1	<0.001	1.045	1.02	1.071
Perceived stress	−0.035	0.022	2.51	1	0.113	0.966	0.925	1.008
Depression	0.095	0.005	388.786	1	<0.001	1.1	1.09	1.111
Anxiety	0.013	0.005	5.963	1	0.015	1.013	1.003	1.024
Psychological pain	0.877	0.023	1429.496	1	<0.001	2.405	2.298	2.517
Latent profile group (ref = Group 1)								
Group 2	0.319	0.05	40.217	1	<0.001	1.376	1.247	1.518
Group 3	0.194	0.097	4.034	1	0.045	1.214	1.005	1.467
Group 4	0.394	0.063	39.64	1	<0.001	1.483	1.312	1.677
Constant	−3.901	0.24	265.243	1	<0.001	0.02		
Model fit indices								
Omnibus test χ^2^			9404.22	17	<0.001			
−2 Log likelihood			26,658.255					
Nagelkerke R^2^			0.37					
Overall classification accuracy			82.50%					

Group 1 = High Resource–Low Victimization; Group 2 = Moderate Resource–Moderate Victimization; Group 3 = Moderate Resource–Polyvictimization; Group 4 = Moderate Resource–Verbal Victimization. CI = confidence interval. Parenting style was entered as dummy variables with “Democratic” as the reference category. The reported OR (1.077) represents the average effect across parenting style categories; this simplification was adopted for model parsimony, with the recognition that the specific estimates for Authoritarian (OR = 1.417, 95% CI: 1.308–1.536), Permissive (OR = 0.987, 95% CI: 0.824–1.183), and Neglectful (OR = 1.174, 95% CI: 1.012–1.363) are presented in Supplementary Table S2 for readers requiring disaggregated results.

## Data Availability

The data supporting the findings of this study are not publicly available due to ethical restrictions protecting participant privacy. De-identified data will be made available upon reasonable request to the corresponding author, subject to approval by our Ethics Committee.

## Supplementary Materials

The following supporting information can be downloaded at: https://www.mdpi.com/article/10.3390/bs16091538/s1, Table S1: Binary Logistic Regression Analysis of Self-Injury Without Psychological Mediators (PHQ-9, GAD-7, TDPPS); Table S2: Logistic Regression Results for Parenting Style (Dummy-Coded, Reference: Democratic).
