# Supplementary material for: Latent Profile Analysis of Resilience, Peer Victimization, and Friendship Quality Configurations Among Chinese Adolescents: Identifying Four Subgroups and Their Self-Injury Prevalence
Source: Behav Sci (Basel). 2026 Sep 1;16(9):1538. doi: 10.3390/bs16091538 (PMC13603898; doi:10.3390/bs16091538)
Supplement: Supplementary file 1 [file behavsci-16-01538-s001.zip › behavsci-4476372-supplementary.pdf]

Supplementary Table S1. Binary Logistic Regression Analysis of Self-Injury Without Psychological Mediators (PHQ-9, GAD-7, TDPPS)

Profile Group	OR	95% CI	p
Moderate Victimization	3.30	3.00 – 3.63	<0.001
Polyvictimization	3.41	2.85 – 4.09	<0.001
Verbal Victimization	2.72	2.43 – 3.04	<0.001

Note: Model adjusted for age, gender, parental marital relationship, parenting style, subjective family economic status, and academic ranking. Reference group: High Resource–Low Victimization. Psychological states (PHQ-9, GAD-7, TDPPS) were not included.

Supplementary Table S2. Logistic Regression Results for Parenting Style (Dummy-Coded, Reference: Democratic)

Parenting Style	OR	95% CI	p	Parenting Style
Authoritarian	1.417	1.308 – 1.536	<0.001	Authoritarian
Permissive	0.987	0.824 – 1.183	0.886	Permissive
Neglectful	1.174	1.012 – 1.363	0.034	Neglectful

Note: Model adjusted for all covariates shown in Table 3. The aggregated OR for parenting style (1.077) reported in Table 3 represents the average effect across categories. Disaggregated estimates are provided here for readers requiring detailed results.
